# Self-Care, Stress Management, and Primary Care: From Salutogenesis and Health Promotion to Mind-Body Medicine

**DOI:** 10.1155/2013/327415

**Published:** 2013-12-31

**Authors:** Tobias Esch, Gregory L. Fricchione, Stefanie Joos, Michael Teut

**Affiliations:** ^1^Harvard Medical School and Division of General Medicine & Primary Care, Beth Israel Deaconess Medical Center, Boston, MA 02215, USA; ^2^Division of Integrative Health Promotion, Coburg University of Applied Sciences, 96450 Coburg, Germany; ^3^Harvard Medical School and Department of Psychiatry, Massachusetts General Hospital, Boston, MA 02114, USA; ^4^Department of General Practice and Health Services Research, Heidelberg University Hospital, 69115 Heidelberg, Germany; ^5^Institute for Social Medicine, Epidemiology and Health Economics, Charité-University Medicine, Berlin, 10117 Berlin, Germany

Stress is a major concern in medicine and the social and health sciences nowadays. In fact, not only are stress-associated ailments and diseases rapidly growing, that is, virtually everywhere in the modern world, but also the cost of treatment and “collateral damage,” for example, in occupational health or the economy as a whole, seems to increase exponentially—for example, see [1, 2]. This may be due to a more recent phenomenon called “burn-out”; however, there is clear evidence that myocardial infarction, stroke, depression, anxiety, and even some disease-prone immune processes, including proinflammation as a common disease denominator that is critically associated with stress [3], are continuously gaining recognition in medicine and bear clear relation upon stress and its (patho)physiology [4]. Thus, preventive and therapeutic options to reduce and prevent stress and, simultaneously, improve stress management skills are strongly needed. Mindfulness-based programs and other mind-body medical and cognitive behavioral strategies to (better) deal with stress are taught and evaluated at many places, including academic medical sites and universities [5]; however, their outcome is usually measured in terms of clinical improvements with disease states, inhibition of disease progression, or cure and relapse prevention.

Since primary care and health promotion can be seen as the first line of defense in medicine and, yet, as setting-oriented “places” where resistance resources, health protection, resiliency, and salutogenesis are facilitated in more complex situations and modalities, it should and will incorporate self-care-oriented means, techniques, and strategies to lower stress at all levels and improve self- or stress management skills of the population as a whole—and of each single individual under treatment. Research in this area is evolving and it focuses on the many aspects that contribute to a healthy and more stress-resilient life-style, hence, looking not only at tangible disease outcomes but also at quality of life, happiness, flourishing, subjective well-being, optimism, and so on—for example, see [6].

With this special issue, we invited investigators to contribute original research as well as review articles that could stimulate the continuing efforts to understand the molecular, (patho)physiological, neurobiological, and, particularly, clinical factors that underlie stress and stress management programs or related interventions. Furthermore, we were interested in articles that focused on any mind-body medical, CAM, cognitive behavioral, or mindfulness-based technique that is believed or said to alleviate stress and the negative outcomes of its burden, that is, examining these interventions in clinical or out-patient and setting-oriented areas, including work-place, occupational and communal health, and, especially of interest, primary care.

As expected, we received a broad array of papers and were able, following rigorous review procedures, to accept a couple of them that still cover the whole picture we had originally thought of. Papers on the effectiveness of a happiness training (positive psychology intervention) or on progressive muscle relaxation (PMR) during lunch breaks in the field of occupational health are now included, as are studies on the applicability and effectiveness of mind-body interventions and more complex programs, including mindfulness-based approaches or self-compassion training, in depression, in anxiety, or in general distress. Various settings are depicted, including the military or school and education. Finally, a focus lies on salutogenesis and resource-orientation in general practice.

We hope this special issue meets the expectations of its readers, as it positively exceeded ours as editors. We are happy to herewith launch this special issue and open the discussion on the aspects raised.

*Tobias Esch* *Tobias Esch* *Gregory L. Fricchione* *Gregory L. Fricchione* *Stefanie Joos* *Stefanie Joos* *Michael Teut* *Michael Teut*
